# Clinical Study of Different Modes of Non-invasive Ventilation Treatment in Preterm Infants With Respiratory Distress Syndrome After Extubation

**DOI:** 10.3389/fped.2020.00063

**Published:** 2020-02-25

**Authors:** Fei Ding, Jingling Zhang, Wenya Zhang, Qian Zhao, Zimei Cheng, Yang Wang, Tao Bo, Hui Wu

**Affiliations:** ^1^Department of Pediatrics, The First Affiliated Hospital of Anhui Medical University, Hefei, China; ^2^Department of Pediatrics, Second Xiangya Hospital, Central South University, Changsha, China; ^3^Department of Pediatrics, First Hospital of Jilin University, Changchun, China

**Keywords:** preterm infants, respiratory distress syndrome, synchronized nasal intermittent positive pressure ventilation, nasal continuous positive airway pressure ventilation, extubation

## Abstract

**Objective:** This study aimed to investigate the clinical efficacy and safety of different non-invasive respiratory support methods in preterm infants with respiratory distress syndrome (RDS) after extubation.

**Methods:** From Oct 2017 to 2018, 120 preterm infants were recruited from the NICUs of three hospitals. They were diagnosed with RDS and required mechanical ventilation. After extubation from mechanical ventilation, these infants were divided into NCPAP group, SNIPPV group and SNIPPV + NCPAP group. The time of non-invasive ventilation, reintubation rate within 72 h, success rate of non-invasive ventilation within 1 week, duration of oxygen therapy, hospital stay and incidence of complications were recorded and compared.

**Results:** Compared with the NCPAP group, the SNIPPV group and the SNIPPV + NCPAP group had significantly higher rate of successful extubation and removal from non-invasive ventilation within 1 week (*P* < 0.05). There were no significant differences among three groups in the time of non-invasive ventilation, time of oxygen therapy, hospital stay or incidence of complications (*P* > 0.05).

**Conclusion:** SNIPPV + NCPAP after mechanical ventilation is a relatively safe and effective ventilation strategy for preterm infants with severe RDS. The use of NCPAP facilitates the turnover of SNIPPV ventilators in developing countries.

## Introduction

With the development of perinatal medicine, the survival rate of preterm infants with extremely/ultra-low birth weight has increased yearly, and the incidence of respiratory distress syndrome (RDS) has also increased over year. Mechanical ventilation can quickly and effectively improve the clinical symptoms of preterm infants with severe RDS, but long-term invasive ventilation may increase the risks for ventilator-associated lung injury and infection. Therefore, the mechanical ventilation in preterm infants with severe RDS should be switched to non-invasive respiratory support as soon as their spontaneous breathing becomes stable and symptoms are improved.

Nasal continuous positive airway pressure ventilation (NCPAP) is most commonly used in clinical practice. However, reintubation is needed in some infants undergoing NCPAP, mainly due to the frequent apnea or severe carbon dioxide (CO_2_) retention. In recent years, synchronized nasal intermittent positive pressure ventilation (SNIPPV) has been increasingly used in China as an enhanced mode of NCPAP. It has been shown that the success rate of extubation after SNIPPV is higher than after traditional NCPAP (1). However, SNIPPV is costly and has not been popularized (2). Theoretically, it is possible to reduce the time of SNIPPV in RDS infants and ease the burden without increasing the failure rate of extubation if the infants are initially administered with respiratory support by SNIPPV after the weaning of mechanical ventilation and then with NCPAP. The present study aimed to explore the efficacy and safety of sequential application of SNIPPV and NCPAP in extubated preterm infants with RDS.

## Materials and Methods

### Study Subjects

Preterm infants who met the inclusion criteria and were born between Oct 2017 and 2018 were recruited from the Departments of Obstetrics of three newborn intensive care units (NICUs). These infants received treatment within half hour after birth. After extubation, the enrolled infants were divided into SNIPPV group, NCPAP group and sequential SNIPPV and NCPAP group (sequential treatment group) (*n* = 40 per group).

The inclusion criteria were as follows: (1) The gestational age was <32 weeks and the birth weight was <1,500 g. (2) The infant met the diagnostic criteria for RDS according to the “Practical Neonatology (4th Edition)” (3). (3) The infant underwent tracheal intubation-mechanical ventilation immediately after being transferred to the NICU. In addition, the infant received non-invasive respiratory support after the withdrawal of mechanical ventilation. (4) Their parents signed the informed consent form before study.

The exclusion criteria were as follows: (1) The infant had congenital malformations of vital organs, birth defects, or genetic/metabolic diseases. (2) The duration of premature membrane rupture was longer than 72 h and there was concomitant intrauterine infection. (3) severe perinatal asphyxia was present. (4) no inform ed consent was obtained before study.

### Methods

NCPAP was performed using the CareFusion TF5000 ventilator, while SNIPPV using the Comen nv8 ventilator. In Comen nv8 ventilator, there is a signal acquisition probe which is connected to the abdomen of infants. The respiratory signals are collected via the probe based on the muscular contraction during the respiration, leading to the synchronous NIPPV.

### Non-invasive Ventilation Parameter Settings

Extubation was performed when the PIP ≤ 18 cmH_2_O, PEEP at 2–4 cmH_2_O, RR ≤ 10 breaths/min, FiO_2_ ≤ 0.4 and normal results on arterial blood gas analysis were present simultaneously. After the withdrawal of mechanical ventilation: (1) In the NCPAP group, the positive end-expiratory pressure (PEEP) was 6 cm H_2_O, and the lowest fraction of inspired oxygen (FiO_2_) was used to achieve a target oxygen saturation of 90–95% (4). NCPAP noninvasive ventilation was weaned when the PEEP was <4 cmH_2_O, FiO_2_ was <0.21 and the results of blood gas analysis were within the acceptable range. (2) In the SNIPPV group, the peak inspiratory pressure (PIP) was 15–25 cmH_2_O, the PEEP was 4–6 cmH_2_O, the respiratory rate (RR) was 15–50 breaths/min, and the lowest FiO_2_ was used to achieve a target oxygen saturation of 90–95%. SNIPPV non-invasive ventilation was weaned when the following conditions were present: a. the PIP was <14 cmH_2_O, PEEP was <4 cmH_2_O, FiO_2_ was <0.3, and RR was <15 breaths/min; b. the infants did not experience apnea and bradycardia; c. the results of arterial blood gas analysis were within the acceptable range (5). (3) In the sequential treatment group, The starting parameters of SNIPPV were the same as in the SNIPPV group, than we shifted to NCPAP when FiO2 <0.35, PIP <20 cmH_2_O and PEEP <6 cmH_2_O were present simultaneously, and then we started the following NCPAP with the same starting parameters of the NCPAP group and considered for weaning the same weaning parameters of the NCPAP group.

An infant was administered with SNIPPV support again if she/he experienced phenomena such as apnea and significant fluctuation in oxygen saturation after weaning of NCPAP or SNIPPV and inhalation of air-oxygen mixture. The time of ventilation was included in the total time of non-invasive ventilation. An infant was administered with endotracheal intubation-mechanical ventilation if she/he still experienced one of the following conditions: (1) progressive dyspnea or frequent apnea with the requirement for balloon-mask positive pressure ventilation; (2) inhaled FiO_2_ > 60% and percutaneous oxygen saturation <85% or blood PaO_2_ <50 mmHg; (3) PaCO_2_ > 60 mmHg with concomitant persistent acidosis (pH <7.20–7.25); (4) no improvement and the presence of pulmonary hemorrhage and tension pneumothorax on chest X-ray examination (6).

### Observations

The observations included: (1) rate of reintubation within 72 h after extubation, success rate of weaning from non-invasive ventilation within 1 week and time of non-invasive ventilation; (2) time of oxygen therapy and time to total enteral nutrition; (3) incidence of complications, including neonatal hypoxic-ischemic encephalopathy (HIE), neonatal feeding intolerance, neonatal pneumonia, bronchopulmonary dysplasia (BPD), retinopathy of prematurity (ROP), and patent ductus arteriosus (PDA); (4) hospital stay and medical cost.

### Statistical Methods

Statistical analysis was performed using the SPSS version 23.0 (Statistical Product and Service Solutions, NY, USA). Data were subjected to normality test. The normally distributed data are expressed as mean ± standard deviation ($\bar{x}$ ± *s*), and compared with one way analysis of variance (ANOVA) among three groups, followed by Student-Newman-Keuls (SNK)-q test. Data with abnormal distribution were compared with the non-parametric test. Qualitative data are expressed as number or percentage, and rates were compared using the χ^2^ test. A value of *P* < 0.05 was considered statistically significant.

## Results

### Baseline Characteristics of Infants in Three Groups

As shown in Tables 1, 2, there were no significant differences among three groups in the sex, gestational age, birth weight, use of pulmonary surfactants, time of mechanical ventilation, and age at breastfeeding initiation (*P* > 0.05).

**Table 1:** Baseline characteristics of infants in three groups (part 1).

**Group**	***n***	**Sex (*M*/*F*, *n*)**	**Gestational age (*$\bar{x}$* ±*s*, week)**	**Birth weight (*$\bar{x}$* ±*s*, *g*)**	**Small for gestational age [*n* (%)]**	**Twins [*n* (%)]**	**Mode of delivery (cesarean section/vaginal delivery)**	**Premature rupture of membranes [*n* (%)]**
NCPAP	40	22/18	29.9 ± 1.4	1.1 ± 0.2	8 (20)	10 (25)	30/10	6 (15)
SNIPPV	40	26/14	29.7 ± 2.3	1.3 ± 0.2	6 (15)	12 (30)	24/16	7 (17.5)
Sequential treatment	40	26/14	29.3 ± 1.8	1.2 ± 0.2	7 (17.5)	8 (20)	26/14	6 (15)
F(χ^2^)		1.128	0.418	1.803	0.346	1.067	2.100	0.125
*P*		0.597	0.661	0.176	0.954	0.628	0.400	0.939

*P refers to three groups of comparison: NCPAP, SNIPPV and Sequetial treatment. The comparison was conducted among the three groups. If P < 0.05, a pairwise comparison was further performed between every two groups. If P > 0.05, it means that there was no statistical difference among them, so no further pairwise comparison would be performed*.

**Table 2:** Baseline characteristics of infants in three groups of infants (part 2).

**Group**	***n***	***In vitro* fertilizatio*n* [*n* (%)]**	**Mother with pregnancy-induced hypertension [*n* (%)]**	**Prenatal use of hormones [*n* (%)]**	Use of PS^*^ [*n* (%)]	**Duration of mechanical ventilation (*$\bar{x}$* ±*s*, day)**	**Age at breast feeding initiation (*$\bar{x}$* ±*s*, day)**
NCPAP	40	8 (20)	12 (30)	38 (95)	38 (95)	7 ± 5	2.9 ± 1.2
SNIPPV	40	10 (25)	14 (35)	39 (97.5)	37 (93.5)	7 ± 5	3.5 ± 1.0
Sequential treatment	40	14 (35)	20 (50)	39 (97.5)	38 (95)	8 ± 5	3.0 ± 1.2
*F*(*χ^2^*)		2.386	3.666	0.517	0.303	0.411	0.915
*P*		0.352	0.164	0.772	0.859	0.665	0.410

* *PS, Pulmonary surfactants. P refers to three groups of comparison: NCPAP, SNIPPV and Sequetial treatment. The comparison was conducted among the three groups. If P < 0.05, a pairwise comparison was further performed between every two groups. If P > 0.05, it means that there was no statistical difference among them, so no further pairwise comparison would be performed*.

### Time of Non-invasive Ventilation, Rate of Reintubation Within 72 h, and Success Rate of Weaning From Non-invasive Ventilation Within One Week

The rate of reintubation within 72 h was significantly lower in the SNIPPV group and sequential treatment group as compared to the NCPAP group, whereas the success rate of weaning from non-invasive ventilation within 1 week was markedly higher in the SNIPPV group and sequential treatment group as compared to the NCPAP group (*P* < 0.05). In addition, no significant difference was noted in the time of non-invasive ventilation among three groups (*P* > 0.05) (Table 3).

**Table 3:** Reintubation rate within 72 h, success rate of weaning from noninvasive ventilation within one week, and time of noninvasive ventilation in three groups.

**Group**	***n***	**Reintubation rate within 72 h [*n* (%)]**	**Success rate of withdrawal of non-invasive ventilation within one week [*n* (%)]**	**Duration of non-invasive ventilation (*$\bar{x}$* ± *s*, day)**
NCPAP	40	10 (25)	16 (40)	6.5 ± 5.9
SNIPPV	40	2 (5)	28 (70)	4.0 ± 1.35
Sequential treatment	40	2 (5)	30 (75)	4.6 ± 1.93
*F*(χ^2^)		10.350	12.127	1.329
*P*		0.007^a^	0.002^a^	0.278^b^

a *Fisher's exact probability method; a refers to two groups of comparison: NCPAP and SNIPPV*.

b *Refers to three groups of comparison: NCPAP, SNIPPV, and Sequetial treatment. The comparison was conducted among the three groups. If P < 0.05, a pairwise comparison was further performed between every two groups. If P > 0.05, it means that there was no statistical difference among them, so no further pairwise comparison would be performed*.

### Incidence of Complications and Mortality

There were no significant differences among three groups in the incidences of HIE, neonatal feeding intolerance, neonatal pneumonia, PDA, BPD and ROP, and mortality (*P* > 0.05) (Table 4).

**Table 4:** Incidence of complications and mortality in three groups [*n* (%)].

**Group**	**HIE**	**Neonatal feeding intolerance**	**Neonatal pneumonia**	**PDA**	**BPD**	**ROP**	**Mortality**
NCPAP	10 (25)	14 (35)	12 (30)	4 (10)	7 (17.5)	6 (15)	4 (10)
SNIPPV	8 (20)	10 (25)	13 (32.5)	3 (7.5)	4 (10)	4 (10)	2 (5)
Sequential treatment	6 (15)	12 (30)	10 (25)	6 (15)	6 (15)	5 (12.5)	2 (5)
χ^2^	1.250	0.952	0.656	1.208	0.959	0.457	1.071
*P*	0.581	0.660	0.882	0.665	0.719	0.940	0.728

*BPD, bronchopulmonary dysplasia; PDA, patent ductus arteriosus; ROP, retinopathy of prematurity; P refers to three groups of comparison: NCPAP, SNIPPV, and Sequetial treatment. The comparison was conducted among the three groups. If P < 0.05, a pairwise comparison was further performed between every two groups. If P > 0.05, it means that there was no statistical difference among them, so no further pairwise comparison would be performed*.

### Time of Oxygen Therapy, Time to Total Enteral Feeding, Hospital Stay, and Medical Cost

As shown in Table 5, there were no statistically significant differences among 3 groups in the time of oxygen therapy, time to total enteral feeding, hospital stay, and medical cost (*P* > 0.05).

**Table 5:** Time of oxygen therapy, time to total enteral feeding, hospital stay and medical cost in three groups.

**Group**	**Time of oxygen therapy (*$\bar{x}$* ±*s*, day)**	**Time to total enteral feeding (*$\bar{x}$* ± *s*, day)**	**Hospital stay (*$\bar{x}$* ±*s*, day)**	**Medical cost (*$\bar{x}$* ±*s*, yuan)**
NCPAP	26.6 ± 11.6	36.2 ± 8.1	44.0 ± 7.9	69945.1 ± 13362.6
SNIPPV	22.8 ± 11.7	37.4 ± 14.8	37.4 ± 14.7	64954.0 ± 16677.5
Sequential treatment	20.9 ± 6.0	36.0 ± 10.3	39.5 ± 11.3	62193.1 ± 16360.1
*F*	1.986	0.015	0.088	1.914
*P*	0.370	0.993	0.784	0.384

*P refers to three groups of comparison: NCPAP, SNIPPV, and Sequetial treatment. The comparison was conducted among the three groups. If P < 0.05, a pairwise comparison was further performed between every two groups. If P > 0.05, it means that there was no statistical difference among them, so no further pairwise comparison would be performed*.

## Discussion

The European Consensus Guidelines on the Management of Respiratory Distress Syndrome (2019) recommend non-invasive ventilation as the best respiratory support for preterm infants with RDS (7). However, approximately half of extremely preterm infants are unable to maintain stable oxygenation under non-invasive ventilation and require endotracheal intubation-mechanical ventilation. The lung of preterm infants is still immature and highly susceptible to external disturbances. Such disturbances may affect the normal development, causing lung diseases (8). To decrease the risk for complications (such as ventilator-associated pneumonia and BPD) and reduce the possibility of long-term oral intubation-induced upper jaw deformity and effects on the tooth development (4), early switching from the mechanical ventilation to the non-invasive ventilation has been advocated, even for extremely preterm infants (9). Therefore, it is of great clinical value to determine the optimal mode for the assisted respiratory support after extubation.

NCPAP is the first mode of non-invasive ventilation used for neonatal respiratory support. It provides a positive airway pressure for infants with spontaneous breathing through a continuous air flow, which enhances the functional residual capacity, reduces the work of breathing, maintains lung expansion, prevents end-expiratory alveolar collapse, and prepares for successful extubation. However, infants who receive NCPAP as the respiratory support after extubation sometimes require reintubation and repeat mechanical ventilation due to some conditions such as apnea, which is usually accompanied by increased risk for complications and elevated medical cost and affects the quality of life of these infants.

Nasal intermittent positive pressure ventilation (NIPPV) provides an intermittent positive-pressure respiratory support at set intervals on the basis of NCPAP. NIPPV provides infants with stable PIP and PEEP and offers stronger respiratory support than NCPAP. NIPPV is a transitional assisted ventilation after extubation, and its efficacy has been confirmed in some randomized controlled studies (10). Xia et al. showed that, compared with NCPAP, NIPPV effectively improved the pulmonary oxygenation, shortened the duration of assisted ventilation, increased the extubation rate, and reduced the incidence of frequent apnea and BPD (11). Lemyre et al. analyzed the results of 10 randomized and semi-randomized trials (12). Their results showed that NIPPV was more effective than NCPAP in reducing the need for reintubation within a week. However, NIPPV had no effect on the chronic lung diseases and mortality. In a retrospective analysis, Bhandari et al. found that NIPPV was more helpful for the weaning from ventilation than NCPAP, and SNIPPV seemed to be as effective as NIPPV (13).

SNIPPV is achieved by adding a synchronous sensor to NIPPV, which renders breathing more suitable to the physiological state of infants. Theoretically, this synchronized mode allows air to efficiently enter the lower respiratory tract and reach the lungs during assisted ventilation. Therefore, SNIPPV has a stronger biological effect than NIPPV. Aghai et al. indicated that SNIPPV could decrease work of breathing (WOB) compared with NCPAP, because SNIPPV can provide positive inspiratory pressure intermittently. The NIPPV mode often causes desynchrony between the ventilator and the infant's spontaneous breathing, which may increase the ventilator-related adverse events, such as apnea and fluctuations of oxygen saturation in infants (14), because asynchronous breaths may induce laryngeal closure, inhibit inspiration, increase abdominal distention, have detrimental effects on blood pressure and cerebral blood flow, and increase WOB. The SNIPPV mode solves the problem of desynchrony. Research from Gizziet et al. showed that SNIPPV could reduce the occurrence of apnea in preterm infants compared with NIPPV and NCPAP (15). Chen et al. found that, as compared to NCPAP, SNIPPV enhanced the success rate of weaning from ventilation, reduced the incidences of apnea and BPD, and shortened the time of oxygen exposure and hospital stay (16).

The Chinese “Expert Consensus on Nasal Intermittent Positive Pressure Ventilation in Preterm Infants (2018)” recommends that NIPPV transition is preferred after extubation of endotracheal tube. After weaning from the NIPPV, infants should be administered with NCPAP, high-flow nasal cannula (HFNC) or nasal cannula oxygen inhalation depending on the disease condition (5). A sequential SNIPPV/NCPAP mode that continued to provide low-parameter NCPAP support after weaning from SNIPPV was established in the present study. Theoretically, this sequential mode may decrease the failure rate of extubation and reduce the risk from ventilator-related lung injury in preterm infants with RDS.

In the present study, the clinical efficacy of three ventilation modes (NCPAP, SNIPPV and sequential SNIPPV/NCPAP) was compared in the treatment of preterm infants with severe RDS after extubation. The results showed that sequential SNIPPV and NCPAP treatment achieved an efficacy similar to SNIPPV alone. Compared with NCPAP alone, the sequential treatment reduced the failure rate of extubation and increased the success rate of weaning from the non-invasive ventilation within 1 week without increasing the risk for complications such as BPD and ROP. There was no significant difference in the time of non-invasive ventilation between SNIPPV group and sequential treatment group. However, the time of SNIPPV was reduced in the sequential treatment group when compared with the SNIPPV group, which reduced the medical cost to a certain extent. Although the medical cost was comparable between two groups, the medical cost was slightly lower in the sequential treatment group than in the SNIPPV group. Compared with the other two groups, the time of oxygen therapy and the time to total enteral nutrition were reduced in the sequential treatment group, although no significant differences were observed. This might be related to the small sample size. Our results should be confirmed by multicenter clinical trials with large sample size. In addition, no patients were followed up, so the short-term/long-term prognosis (such as long-term lung function and neurodevelopmental outcome) was unclear.

In summary, the sequential SNIPPV/NCPAP mode can safely and effectively facilitate the weaning from invasive mechanical ventilation in preterm infants with RDS without increasing the risk for complications and medical cost.

## Data Availability Statement

The datasets generated for this study are available on request to the corresponding author.

## Ethics Statement

The studies involving human participants were reviewed and approved by Clinical medical research ethics committee of The First Affiliated Hospital of Anhui Medical University. Written informed consent to participate in this study was provided by the participants' legal guardian/next of kin.

## Author Contributions

YW, TB, and HW contributed to the conception and design of this study. FD, JZ, and WZ organized the data. QZ and ZC performed the statistical analysis. FD, JZ, and WZ drafted the manuscript. QZ and ZC wrote sections of the manuscript. All authors contributed to manuscript revision, read and approved the submitted version.

### Conflict of Interest

The authors declare that the research was conducted in the absence of any commercial or financial relationships that could be construed as a potential conflict of interest.
